# Type 2 Diabetes Mellitus Versus Adverse In-Hospital Outcomes After Partial or Radical Nephrectomy

**DOI:** 10.1245/s10434-026-19225-9

**Published:** 2026-02-27

**Authors:** Maximilian Filzmayer, Michele Nicolazzini, Calogero Catanzaro, Federico Polverino, Michele Petix, Leonardo Quarta, Jordan A. Goyal, Alessandro Volpe, Riccardo Schiavina, Nicola Longo, Gennaro Musi, Alberto Briganti, Shahrokh F. Shariat, Mike Wenzel, Marina Kosiba, Clara Humke, Fred Saad, Felix K.-H. Chun, Pierre I. Karakiewicz

**Affiliations:** 1 Cancer Prognostics and Health Outcomes Unit, Division of Urology, University of Montreal Health Center, Montreal, Canada; 2 Department of Urology, Goethe University Frankfurt, University Hospital, Frankfurt am Main, Germany; 3 Division of Urology, Department of Translational Medicine, University of Eastern Piedmont, Maggiore della Carità Hospital, Novara, Italy; 4 Division of Urology, Department of Oncology, University of Turin, Orbassano, Italy; 5 Department of Urology, University of Bologna, St. Orsola-Malpighi Hospital, Bologna, Italy; 6 Department of Neurosciences, Science of Reproduction and Odontostomatology, University of Naples Federico II, Naples, Italy; 7 Department of Urology, IEO European Institute of Oncology, IRCCS, Milan, Italy; 8 Department of Oncology and Haemato-Oncology, Università degli Studi di Milano, Milan, Italy; 9 Division of Experimental Oncology/Unit of Urology, URI, Urological Research Institute, IRCCS San Raffaele Scientific Institute, Milan, Italy; 10 Vita-Salute San Raffaele University, Milan, Italy; 11 Department of Urology, Comprehensive Cancer Center, Medical University of Vienna, Vienna, Austria; 12 Department of Urology, Weill Cornell Medical College, New York, NY USA; 13 Department of Urology, University of Texas Southwestern Medical Center, Dallas, TX USA; 14 Hourani Center of Applied Scientific Research, Al-Ahliyya Amman University, Amman, Jordan

**Keywords:** NIS, Nephrectomy, Kidney cancer, Type 2 diabetes mellitus, Insulin-dependence

## Abstract

**Background:**

The effect of insulin-dependence in type 2 diabetes mellitus (T2DM) on adverse in-hospital outcomes after partial (PN) or radical nephrectomy (RN) is unknown.

**Patients and Methods:**

Descriptive statistics, propensity score matching (PSM), and multivariable logistic regression were applied to the National Inpatient Sample (2004–2019) patients with kidney cancer who underwent nephrectomy. T2DM was stratified between insulin-dependent (ID) and noninsulin-dependent (NID) subtypes.

**Results:**

In 31,909 patients treated with PN, rates of ID-T2DM versus NID-T2DM were 3.5% versus 20.0%, and 3.4% versus 20.9% in 57,029 patients treated with RN. During the study period, ID-T2DM rates increased from 0.02% to 5.4% (270-fold) in PN and from 0.3% to 6.3% (21-fold) in RN. NID-T2DM rates increased from 17.4% to 20.2% (1.2-fold) in PN and from 17.0% to 21.6% (1.3-fold) in RN. After PSM, patients with ID-T2DM who underwent PN (1109 versus 5545 nondiabetic controls) exhibited higher rates of adverse in-hospital outcomes with significant increases in six examined categories (7.9–2.3%; OR 1.6–1.3). ID-T2DM had a weaker effect (6.0–0.6%; OR 3.0–1.2) in patients treated with RN (1961 versus 3922 controls). Finally, NID-T2DM exerted a modest effect (3.5–2.5%, OR 1.4–1.2) in patients treated with PN (6400 versus 6400 controls) and the weakest effect (2.6–0.8%, OR 1.2–1.1) in patients treated with RN (11,924 versus 11,924 controls).

**Conclusions:**

Although ID-T2DM is relatively rare, its rates increased drastically over time. ID-T2DM was most strongly associated with adverse in-hospital outcomes in patients treated with PN, both in absolute and relative terms. Therefore, patients with ID-T2DM undergoing PN may represent a particularly relevant target population for perioperative management optimization with respect to surgical risk.

**Supplementary Information:**

The online version contains supplementary material available at 10.1245/s10434-026-19225-9.

Rates of type 2 diabetes mellitus (T2DM) in North America are rising and many patients with kidney cancer undergoing partial nephrectomy (PN) or radical nephrectomy (RN) may harbor T2DM.^1–3^ Previous studies in the field of diabetology had identified insulin-dependent (ID) T2DM as a risk factor for poor long-term outcomes compared with noninsulin-dependent (NID) T2DM, including significantly higher all-cause and cardiovascular mortality.^4,5^ Furthermore, emerging evidence in orthopedic surgery indicates that insulin-dependence may represent an important modifier of surgical risk.^6,7^ It is unknown to what extent presence of T2DM and its subtype regarding insulin-dependence may increase the rates of adverse in-hospital outcomes after PN or RN.^8–10^

We addressed this knowledge gap using a large-scale contemporary cohort of kidney cancer patients who underwent PN or RN derived from the National Inpatient Sample (NIS) database. Given the structure and purpose of the NIS database, which captures events during the index hospitalization, the present analysis focuses on adverse in-hospital outcomes as clinically relevant, short-term end points.^11^ These outcomes complement long-term oncologic and functional results by identifying perioperative risk and potentially modifiable vulnerabilities in specific subgroups for whom targeted perioperative optimization strategies may be most effective. We hypothesized that patients with T2DM may be at a disadvantage when adverse in-hospital outcomes after PN or RN represent the end points of interest. In addition, we hypothesized that there might be a dose-response effect according to ID or NID status.

## Patients and Methods

### Data Source and Study Population

Relying on discharge data from the NIS 2004–2019, we assessed perioperative complications, length of stay, and in-hospital mortality of patients with kidney cancer treated with PN or RN within the USA. NIS is a set of longitudinal hospital inpatient databases included in the Healthcare Cost and Utilization Project (HCUP).^11^

All diagnoses and procedures were coded using the International Classification of Disease (ICD) 9th Revision (ICD-9) Clinical Modification, ICD 10th revision (ICD-10) Clinical Modification or ICD-10 Procedure Coding System. In accordance with previously reported methodology, we identified patients aged ≥ 18 years with primary kidney cancer who were treated with PN or RN.^12–14^ Patients were stratified according to the diagnosis of T2DM and insulin-dependence status.^7,15^ Patients with other or unknown forms of diabetes were excluded.

### Outcomes of Interest

Our primary end point was adverse in-hospital outcomes, consisting of overall complications, intraoperative complications, postoperative complications, critical care therapies, bleeding complications, blood transfusions, cardiovascular complications, pulmonary complications, genitourinary complications, gastrointestinal complications, infectious complications, and neurologic complications, identified by ICD-9 and ICD-10 codes in previously established methodology.^12–15^ In addition, in-hospital mortality and prolonged length of stay (LOS; exceeding the third quartile for the cohort; PN: > 4 days; RN: > 6 days) were assessed.^12^ We relied on the coding algorithms by Quan et al. to define comorbidities using ICD-9 and ICD-10 codes.^16^ The Deyo adaption of the Charlson Comorbidity Index (CCI) was applied, excluding T2DM from the calculation.^17^ The covariables included age (in years), sex (male versus female), ethnicity (Caucasian versus African American versus Hispanic versus other), CCI (0–1 versus ≥ 2), obesity (yes versus no), year of surgery, minimally invasive surgery (yes versus no), and hospital size (large versus medium versus small; defined by bed count specific to region, urban or rural location, and teaching-hospital status^11^). According to the World Health Organization, obesity was defined as a body mass index exceeding 30 kg/m^2^.^18^

### Statistical Analyses

For continuously coded variables, medians and interquartile ranges (IQRs) were calculated. For categorically coded variables, absolute frequencies and proportions were determined. Wilcoxon rank-sum and Pearson chi-squared tests assessed differences between patients with ID-T2DM and NID-T2DM compared with nondiabetic patients. The estimated annual percentage changes (EAPC) of ID-T2DM and NID-T2DM rates were calculated using least-squares linear regression. To minimize bias and confounding, we applied propensity score matching (PSM) in four separate analytical steps. First, PSM was applied between patients with ID-T2DM who underwent PN and nondiabetic PN controls (comparison A). Second, it was reapplied between patients with ID-T2DM who underwent RN and nondiabetic RN controls (comparison B). Third, it was reapplied between patients with NID-T2DM who underwent PN and nondiabetic PN controls (comparison C). Fourth, it was reapplied between patients with NID-T2DM who underwent RN and nondiabetic RN controls (comparison D). PSM relied on patient age, sex, ethnicity, CCI, obesity status, minimally invasive procedure, and year of surgery. Different matching ratios were used across the four comparisons to maximize statistical efficiency while preserving adequate covariate balance, given the substantial differences in available sample sizes between ID-T2DM, NID-T2DM, and nondiabetic patients within PN and RN cohorts. Multivariable logistic regression models predicting adverse in-hospital outcomes and adjusting for patient age, sex, ethnicity, CCI, obesity status, year of surgery, minimally invasive procedure, and hospital size were fitted. The analysis followed the NIS reporting guidelines. Therefore, absolute counts for samples < 11 could not be reported.^11^ R software was used for statistical computing and graphics (R version 4.4.1, The R Foundation for Statistical Computing, Vienna, Austria). All tests were two-sided with significance set at *p* < 0.05.

## Results

### Descriptive Characteristics of the Study Population

Within the NIS (2004–2019), we identified 31,909 patients treated with PN for kidney cancer and 57,029 patients treated with RN for kidney cancer, respectively (Table 1). Of the patients who underwent PN, 1109 (3.5%) had ID-T2DM and 6400 (20.0%) had NID-T2DM. Of the patients who underwent RN, 1961 (3.4%) had ID-T2DM and 11,924 (20.9%) had NID-T2DM.

**Table 1:** Descriptive characteristics of patients treated with partial or radical nephrectomy, stratified according to type 2 diabetes mellitus and insulin dependence, before propensity score matching

Characteristic	Partial Nephrectomy *n* = 31,909					Radical Nephrectomy *n* = 57,029				
	ID-T2DM patients *n* = 1109 (3.5%)	*p*-value^1^	NID-T2DM patients *n* = 6400 (20.0%)	*p*-value^1^	nondiabetic patients *n* = 24,400 (76.5%)	ID-T2DM patients *n* = 1961 (3.4%)	*p*-value^1^	NID-T2DM patients *n* = 11,924 (20.9%)	*p*-value^1^	nondiabetic patients *n* = 43,144 (75.7%)
Age, median (IQR), in years	62 (56, 69)	**<0.001**	63 (57, 70)	**<0.001**	59 (50, 68)	65 (57, 71)	**<0.001**	66 (58, 73)	**<0.001**	62 (53, 72)
Male sex, *n* (%)	654 (59.0%)	**0.029**	3929 (61.4%)	0.2	15,182 (62.2%)	1169 (59.6%)	**0.048**	7431 (62.3%)	0.3	26,676 (61.8%)
Ethnicity, *n* (%)		**<0.001**		**<0.001**			**<0.001**		**<0.001**	
Caucasian	740 (66.7%)		4442 (69.4%)		18,626 (76.3%)	1267 (64.6%)		8479 (71.1%)		33,516 (77.7%)
African American	181 (16.3%)		835 (13.0%)		2432 (10.0%)	341 (17.4%)		1396 (11.7%)		4120 (9.5%)
Hispanic	124 (11.2%)		678 (10.6%)		1908 (7.8%)	245 (12.5%)		1340 (11.2%)		3444 (8.0%)
Other	64 (5.8%)		445 (7.0%)		1434 (5.9%)	108 (5.5%)		709 (5.9%)		2064 (4.8%)
Charlson Comorbidity Index^2^, *n* (%)		**<0.001**		**<0.001**			**<0.001**		**<0.001**	
0–1	695 (62.7%)		4754 (74.3%)		20,708 (84.9%)	1024 (52.2%)		8068 (67.7%)		34,737 (80.5%)
≥ 2	414 (37.3%)		1646 (25.7%)		3692 (15.1%)	937 (47.8%)		3856 (32.3%)		8407 (19.5%)
Obesity^3^, *n* (%)	421 (38.0%)	**<0.001**	1883 (29.4%)	**<0.001**	3737 (15.3%)	698 (35.6%)	**<0.001**	3045 (25.5%)	**<0.001**	5471 (12.7%)
Year of surgery, median (IQR)	2016 (2012, 2018)	**<0.001**	2014 (2010, 2017)	**0.002**	2013 (2010, 2017)	2015 (2011, 2017)	**<0.001**	2012 (2008, 2016)	**<0.001**	2012 (2008, 2016)
Minimally invasive procedure, *n* (%)	608 (54.8%)	**0.002**	3118 (48.7%)	0.1	12,189 (50.0%)	754 (38.4%)	**<0.001**	3651 (30.6%)	0.9	13,214 (30.6%)
Hospital size^4^, *n* (%)		0.4		0.5			0.2		0.5	
Large	729 (65.7%)		4293 (67.1%)		16,412 (67.3%)	1235 (63.0%)		7698 (64.6%)		28,060 (65.0%)
Medium	241 (21.7%)		1384 (21.6%)		5252 (21.5%)	492 (25.1%)		2834 (23.8%)		10,208 (23.7%)
Small	139 (12.5%)		723 (11.3%)		2736 (11.2%)	234 (11.9%)		1392 (11.7%)		4876 (11.3%)

^1^Wilcoxon rank-sum test, Pearson's chi-squared test, Reference: nondiabetic patients.

^2^The point contribution of CCI due to T2DM was subtracted.

^3^Body mass index > 30 kg/m^2^.

^4^Defined by bed count; specific to region, urban or rural location and teaching-hospital status.

*ID* Insulin-dependent, *IQR* interquartile range, *NID* Non-insulin-dependent, *T2DM* type 2 diabetes mellitus

Statistically significant values are shown in bold (*p* < 0.05).

**Fig. 1:**
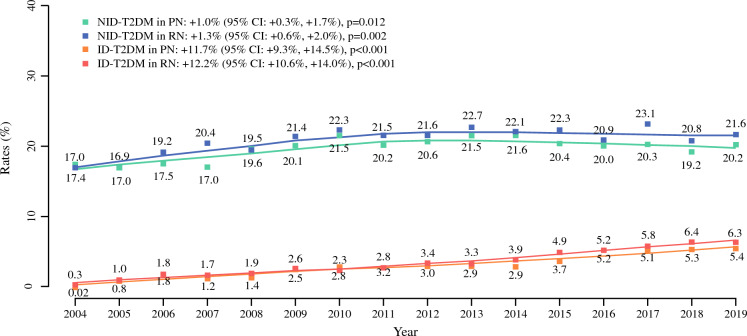
Estimated annual percentage change (EAPC) of type 2 diabetes mellitus (T2DM) stratified between insulin-dependent (ID) and non-insulin-dependent (NID) in kidney cancer patients treated with partial (PN) or radical nephrectomy (RN) within the National Inpatient Sample (NIS) from 2004 to 2019

From 2004 to 2019, the rates of ID-T2DM increased 270-fold from 0.02% to 5.4% in patients treated with PN (EAPC: +11.7%, *p* < 0.001, Fig. 1) and 21-fold from 0.3% to 6.3% in patients treated with RN (EAPC: +12.2%, *p* < 0.001). The rates of NID-T2DM increased 1.2-fold from 17.4% to 20.2% in patients treated with PN (EAPC: +1.0%, *p* = 0.01) and 1.3-fold from 17.0% to 21.6% in patients treated with RN (EAPC: +1.3%, *p* < 0.01).

Before PSM and across all four groups of interest (comparison A: ID-T2DM in PN; comparison B: ID-T2DM in RN; comparison C: NID-T2DM in PN; comparison D: NID-T2DM in RN), diabetic patients were older, more frequently African American or Hispanic, more often obese, and more likely to present with a CCI ≥ 2 compared with nondiabetic patients (Table 1). These differences were more pronounced among patients with ID-T2DM, for example obesity occurred in 15.3% of nondiabetic patients versus 29.4% of patients with NID-T2DM versus 38.0% patients with ID-T2DM. Compared with RN, patients with PN were younger, less frequently exhibited a CCI ≥ 2 and were more frequently approached with a minimally invasive procedure.

### Propensity Score Matching

We applied PSM in four separate analytical steps. First, 1109 of 1109 patients with ID-T2DM who underwent PN (100%) were matched in 1:5 fashion to 5545 of 24,400 nondiabetic PN controls (22.7%; comparison A). Second, 1961 of 1961 patients with ID-T2DM who underwent RN (100%) were matched in 1:2 fashion to 3922 of 43,144 nondiabetic RN controls (9.1%; comparison B). Third, 6400 of 6400 patients with NID-T2DM who underwent PN (100%) were matched in 1:1 fashion to 6400 of 24,400 nondiabetic PN controls (26.2%; comparison C). Fourth, 11,924 of 11,924 patients with NID-T2DM who underwent RN (100%) were matched in 1:1 fashion to 11,924 of 43,144 nondiabetic RN controls (27.6%; comparison D). After PSM, no statistically significant or clinically meaningful differences between diabetic patients and their nondiabetic controls remained (Supplementary Table S1).

### ID-T2DM versus Adverse In-Hospital Outcomes after PN

Patients with ID-T2DM who underwent PN exhibited significantly higher rates in six of 14 examined categories compared with their nondiabetic counterparts (Table 2; comparison A). Absolute increases in rates of examined complications ranged from 7.9% to 2.3% (all *p* < 0.01). These absolute increases translated into relative increases that ranged from 1.6- to 1.3-fold (all *p* < 0.05). The strongest relative increases applied to genitourinary complications (OR 1.6), prolonged LOS (OR 1.5), and blood transfusions (OR 1.5).

**Table 2:** Adverse in-hospital outcomes after partial or radical nephrectomy, stratified according to presence of insulin-dependent type 2 diabetes mellitus or absence of any type 2 diabetes mellitus, after propensity score matching

Characteristic	Partial nephrectomy *n* = 6654				Radical nephrectomy *n* = 5883			
	ID-T2DM patients *n*=1109 (100%)	nondiabetic patients^1^ *n*= 5545 (22.7%)	Difference (*Δ*)^2^	Multivariable LRM OR (95% CI)^3^	ID-T2DM patients *n*= 1961 (100%)	nondiabetic patients^1^ *n*= 3922 (9.1%)	Difference (*Δ*)^2^	Multivariable LRM OR (95% CI)^3^
Overall complications, *n* (%)	423 (38.1%)	1807 (32.6%)	+5.5%***	1.3 (1.1–1.5)***	878 (44.8%)	1552 (39.6%)	+5.2%***	1.2 (1.1–1.4)***
Intraoperative complications, *n* (%)	56 (5.0%)	315 (5.7%)	−0.7%	0.9 (0.6–1.2)	153 (7.8%)	328 (8.4%)	−0.6%	1.0 (0.8–1.3)
Postoperative complications, *n* (%)	419 (37.8%)	1752 (31.6%)	+6.2%***	1.3 (1.1–1.5)***	859 (43.8%)	1484 (37.8%)	+6.0%***	1.3 (1.1–1.4)***
Critical care therapies, *n* (%)	22 (2.0%)	125 (2.3%)	−0.3%	0.9 (0.5–1.3)	68 (3.5%)	168 (4.3%)	−0.8%	0.8 (0.6–1.1)
Bleeding complications, *n* (%)	19 (1.7%)	108 (1.9%)	−0.2%	0.9 (0.5–1.4)	45 (2.3%)	121 (3.1%)	−0.8%	0.8 (0.5–1.1)
Blood transfusion, *n* (%)	105 (9.5%)	349 (6.3%)	+3.2%***	1.5 (1.2–2.0)***	216 (11.0%)	365 (9.3%)	+1.7%*	1.3 (1.1–1.6)**
Cardiovascular complications, *n* (%)	100 (9.0%)	457 (8.2%)	+0.8%	1.2 (0.9–1.5)	225 (11.5%)	381 (9.7%)	+1.8%*	1.1 (0.9–1.3)
Pulmonary complications, *n* (%)	108 (9.7%)	412 (7.4%)	+2.3%**	1.3 (1.0–1.6)*	176 (9.0%)	368 (9.4%)	−0.4%	1.0 (0.8–1.2)
Genitourinary complications, *n* (%)	226 (20.4%)	780 (14.1%)	+6.3%***	1.6 (1.3–1.9)***	468 (23.9%)	737 (18.8%)	+5.1%***	1.3 (1.2–1.5)***
Gastrointestinal complications, *n* (%)	93 (8.4%)	398 (7.2%)	+1.2%	1.2 (0.9–1.5)	190 (9.7%)	416 (10.6%)	−0.9%	0.9 (0.8–1.1)
Infectious complications, *n* (%)	< 11 (< 1.0%)	56 (1.0%)	NA	0.6 (0.2–1.2)	18 (0.9%)	45 (1.1%)	−0.2%	0.8 (0.4–1.3)
Neurologic complications, *n* (%)	< 11 (< 1.0%)	22 (0.4%)	NA	1.4 (0.5–3.2)	17 (0.9%)	11 (0.3%)	+0.6%***	3.0 (1.4–6.6)**
In-hospital mortality, *n* (%)	< 11 (< 1.0%)	19 (0.3%)	NA	NA	<11 (<0.6%)	22 (0.6%)	NA	0.7 (0.3–1.6)
Prolonged length of stay^4^, *n* (%)	335 (30.2%)	1234 (22.3%)	+7.9%***	1.5 (1.3–1.8)***	371 (18.9%)	684 (17.4%)	+1.5%*	1.2 (1.0–1.4)*

^1^Propensity score matching relied on patient age, sex, ethnicity, Charlson Comorbidity Index, obesity status, year of surgery, minimally invasive procedure and hospital size.

^2^Wilcoxon rank sum test, Pearson’s chi-square test.

^3^Reference: matched nondiabetic patients; adjusted for patient age, sex, ethnicity, Charlson Comorbidity Index, obesity status, year of surgery, minimally invasive procedure and hospital size.

^4^Exceeding the third quartile for the cohort (> 4 days for partial and > 6 days for radical nephrectomy).

*CI* confidence interval, *ID* Insulin-dependent, *LRM* logistic regression model, *NA* not available, *OR* odds ratio, *T2DM* type 2 diabetes mellitus

* *p* < 0.05, ** *p* < 0.01, *** *p* < 0.001; statistically significant values are shown in bold (*p* < 0.05).

### ID-T2DM versus Adverse In-Hospital Outcomes after RN

Patients with ID-T2DM who underwent RN exhibited significantly higher rates in 7 of 14 examined categories compared with their nondiabetic counterparts (comparison B). Absolute increases in rates of examined complications ranged from 6.0% to 1.5% and translated into relative increases that ranged from 3.0-fold (neurologic complications) to 1.2-fold (prolonged LOS; all *p* < 0.05).

### NID-T2DM versus Adverse In-Hospital Outcomes after PN

Patients with NID-T2DM who underwent PN exhibited significantly higher rates in 5 of 14 examined categories compared with their nondiabetic counterparts (Table 3; comparison C). Absolute increases in rates of examined complications ranged from 3.5% to 2.5% and translated into relative increases that ranged from 1.4-fold (blood transfusions) to 1.2-fold (genitourinary complications; all *p* < 0.001).

**Table 3:** Adverse in-hospital outcomes after partial or radical nephrectomy, stratified according to presence of non-insulin-dependent type 2 diabetes mellitus or absence of any type 2 diabetes mellitus, after propensity score matching

Characteristic	Partial nephrectomy *n* = 12,800				Radical nephrectomy *n* = 23,848			
	NID-T2DM patients *n* = 6400 (100%)	nondiabetic patients^1^ *n* = 6400 (26.2%)	Difference (*Δ*)^2^	Multivariable LRM OR (95% CI)^3^	NID-T2DM patients *n* = 11,924 (100%)	nondiabetic patients^1^ *n* = 11,924 (27.6%)	Difference (*Δ*)^2^	Multivariable LRM OR (95% CI)^3^
Overall complications, *n* (%)	2247 (35.1%)	2033 (31.8%)	+3.3%***	1.2 (1.1–1.3)***	4770 (40.0%)	4459 (37.4%)	+2.6%***	1.1 (1.1–1.2)***
Intraoperative complications, *n* (%)	447 (7.0%)	480 (7.5%)	−0.5%	0.9 (0.8–1.1)	1102 (9.2%)	1029 (8.6%)	+0.6%	1.1 (1.0–1.2)
Postoperative complications, *n* (%)	2170 (33.9%)	1969 (30.8%)	+3.1%***	1.2 (1.1–1.3)***	4614 (38.7%)	4306 (36.1%)	+2.6%***	1.1 (1.1–1.2)***
Critical care therapies, *n* (%)	139 (2.2%)	147 (2.3%)	−0.1%	0.9 (0.7–1.2)	452 (3.8%)	421 (3.5%)	+0.3%	1.1 (0.9–1.2)
Bleeding complications, *n* (%)	119 (1.9%)	152 (2.4%)	−0.5%	0.8 (0.6–1.0)	338 (2.8%)	317 (2.7%)	+0.1%	1.1 (0.9–1.2)
Blood transfusion, *n* (%)	581 (9.1%)	425 (6.6%)	+2.5%***	1.4 (1.2–1.6)***	1389 (11.6%)	1173 (9.8%)	+1.8%***	1.2 (1.1–1.3)***
Cardiovascular complications, *n* (%)	427 (6.7%)	401 (6.3%)	+0.4%	1.1 (1.0–1.3)	946 (7.9%)	844 (7.1%)	+0.8%*	1.1 (1.0–1.2)*
Pulmonary complications, *n* (%)	507 (7.9%)	500 (7.8%)	+0.1%	1.0 (0.9–1.1)	1168 (9.8%)	1060 (8.9%)	+0.9%*	1.1 (1.0–1.2)*
Genitourinary complications, *n* (%)	1029 (16.1%)	870 (13.6%)	+2.5%***	1.2 (1.1–1.4)***	2297 (19.3%)	1991 (16.7%)	+2.6%***	1.2 (1.1–1.3)***
Gastrointestinal complications, *n* (%)	528 (8.3%)	505 (7.9%)	+0.4%	1.1 (0.9–1.2)	1260 (10.6%)	1312 (11.0%)	−0.4%	1.0 (0.9–1.0)
Infectious complications, *n* (%)	45 (0.7%)	64 (1.0%)	−0.3%	0.7 (0.5–1.0)	143 (1.2%)	146 (1.2%)	0.0%	1.0 (0.8–1.2)
Neurologic complications, *n* (%)	24 (0.4%)	19 (0.3%)	+0.1%	1.3 (0.7–2.4)	60 (0.5%)	48 (0.4%)	+0.1%	1.3 (0.9–1.8)
In-hospital mortality, *n* (%)	17 (0.3%)	25 (0.4%)	−0.1%	0.7 (0.4–1.3)	89 (0.7%)	68 (0.6%)	+0.1%	1.3 (1.0–1.8)
Prolonged length of stay^4^, *n* (%)	1795 (28.0%)	1571 (24.5%)	+3.5%***	1.2 (1.1–1.3)***	2087 (17.5%)	1925 (16.1%)	+1.4%**	1.1 (1.0–1.2)**

^1^Propensity score matching relied on patient age, sex, ethnicity, Charlson Comorbidity Index, obesity status, year of surgery, minimally invasive procedure and hospital size.

^2^Wilcoxon rank sum test, Pearson’s chi-square test.

^3^Reference: matched nondiabetic patients; adjusted for patient age, sex, ethnicity, Charlson Comorbidity Index, obesity status, year of surgery, minimally invasive procedure and hospital size.

^4^Exceeding the third quartile for the cohort (> 4 days for partial and > 6 days for radical nephrectomy).

*CI* confidence interval, *LRM* logistic regression model, *NID* Non-insulin-dependent, *OR* odds ratio, *T2DM* type 2 diabetes mellitus

* *p* < 0.05, ** *p* < 0.01, *** *p* < 0.001; statistically significant values are shown in bold (*p* < 0.05).

### NID-T2DM versus Adverse In-Hospital Outcomes after RN

Patients with NID-T2DM who underwent RN exhibited significantly higher rates in 7 of 14 examined categories compared with their nondiabetic counterparts (comparison D). Absolute increases in rates of examined complications ranged from 2.6% to 0.8% and translated into relative increases that ranged from 1.2-fold (genitourinary complications) to 1.1-fold (cardiovascular complications; all *p* < 0.05).

## Discussion

The effect of T2DM—either NID or ID—on adverse in-hospital outcomes in patients undergoing PN or RN is unknown. We investigated this knowledge gap and observed several noteworthy findings.

First, our investigation included 31,909 patients with kidney cancer who underwent PN and 57,029 who underwent RN. Among them, diagnosis of NID-T2DM was relatively frequent in both patients who underwent PN (20.0%) and RN (20.9%). Conversely, the rate of ID-T2DM was substantially lower (3.5% in PN and 3.4% in RN). These observations are consistent with epidemiological data reporting a prevalence of 29.2% for T2DM regardless of insulin-dependence status among USA citizens older than 65 years.^2^ They also agree with two previous smaller-scale studies, where patients with kidney cancer undergoing nephrectomy were stratified according to presence or absence of diabetes. In one of the two studies, Hua et al. examined 74 diabetic versus 769 nondiabetic patients.^8^ Conversely, Shah et al. examined 478 diabetic versus 1,346 nondiabetic patients.^9^ Unfortunately, neither study stratified according to ID versus NID, and this stratification is key when examining the effect of T2DM, as will be shown in the paragraphs below. In addition, the previous studies did not formally address a battery of adverse in-hospital outcomes using standardized coding schemes. In that context, the current study relies on the largest sample size and is capable of providing the most robust observations addressing ID-T2DM and NID-T2DM as surgical risk factor in patients undergoing PN and RN.

Second, to the best of our knowledge, we provide the first report of temporal trends in rates of either ID-T2DM or NID-T2DM in patients undergoing nephrectomy. The rates increased significantly over time, with a drastically steeper slope for ID-T2DM in patientes treated with PN (270-fold; from 0.02% to 5.4%) and, to a lesser extent, in patients treated with RN (21-fold; from 0.3% to 6.3%). Conversely, the increase of NID-T2DM was moderate at best, as evidenced by 1.2-fold (from 17.4% to 20.2%) increase in patients treated with PN and 1.3-fold (from 17.0% to 21.6%) increase in patients treated with RN. These differences emphasize and validate the need for stratification between ID-T2DM and NID-T2DM in addition to stratification between PN and RN, as was done in the current study. The temporal trend observations regarding rates of ID-T2DM and NID-T2DM made in the current study cannot be compared with previous studies addressing T2DM in patients undergoing nephrectomy or other surgical interventions. However, ID-T2DM rates recorded in the current study are consistent with general USA population data, where an increase in ID-T2DM rates from 0.7% in 2009 to 18.4% in 2018 (26-fold) was reported.^19^ This trend may be attributed to the well-described increase of metabolic syndrome in the USA, that according to experts is mainly driven by increased consumption of ultra-processed foods, sugary drinks, larger portion sizes, and physical inactivity.^19,20^

Third, we identified important differences that distinguished diabetic from nondiabetic patients. Diabetic patients were older, more frequently African American or Hispanic, more frequently obese, and more frequently exhibited a CCI ≥ 2. Differences were more pronounced in patients with ID-T2DM than in patients with NID-T2DM. These findings are consistent with the current literature, as well as with epidemiological data from the USA.^2,21,22^ Moreover, patients treated with PN are well known to be different from patients treated with RN.^12,13,23^ For that purpose, PSM was applied according to T2DM subtypes (ID versus NID) in separate groups that distinguished patients who underwent PN from those who underwent RN. Different matching ratios were used due to sample size differences across the four groups of interest (comparison A: ID-T2DM in PN; comparison B: ID-T2DM in RN; comparison C: NID-T2DM in PN; comparison D: NID-T2DM in RN). PSM methodology was not applied in the previous reports.^8,9^

Fourth, after PSM, multivariable logistic regression models were fitted to first test the effect of ID-T2DM versus no T2DM in patients who underwent PN (comparison A). Subsequently, the same models were refitted in patients who underwent RN (comparison B). Thereafter, separate multivariable logistic regression models testing the effect of NID-T2DM versus no T2DM were applied to patients who underwent PN (comparison C) and then refitted in patients who underwent RN (comparison D). The analyses indicated that presence of ID-T2DM in PN patients has a strong effect on adverse in-hospital outcomes. Specifically, ID-T2DM in patients treated with PN resulted in absolute increases that ranged from 6.3% to 2.3%, when specific complications were examined. An even higher absolute increase of 7.9% was recorded when length of stay greater than 4 days presented the end point of interest. These absolute rates translated into relative rates that ranged from 1.6 to 1.3. The three most affected examined categories were genitourinary complications (OR 1.6), prolonged LOS (OR 1.5), and blood transfusions (OR 1.5). When the same models were applied to patients who underwent RN, a moderate effect of ID-T2DM was recorded (6.0–0.6%; OR 3.0–1.2). Conversely, patients treated with PN exhibited a modest effect, when the same testing was performed between NID-T2DM and no T2DM (3.5–2.5%; OR 1.4–1.2). The weakest effect was observed in patients with NID-T2DM who underwent RN, while most observed associations were characterized by relatively small absolute risk differences (2.6–0.8%; OR 1.2–1.1). It may be postulated that the stronger effect at PN may in part reflect the higher technical complexity. In contrast to RN, PN involves precise tumor resection and renal reconstruction, which likely increases the susceptibility of diabetic patients to surgical complications, particularly bleeding and genitourinary complications.^9,24^ However, alternative or complementary explanations should be acknowledged including unmeasured tumor-related factors such as anatomical complexity, which are not captured in the NIS and may be particularly relevant in PN.

Finally, in all four examined subgroups, no significant association was observed between either ID-T2DM or NID-T2DM on in-hospital mortality or use of critical care therapies. This finding suggests that while predominantly ID-T2DM and to a much lesser extent NID-T2DM contributes to postoperative morbidity, it does not appear to translate into escalated therapeutic intensity or short-term mortality.

Taken together, these robust observations suggest that ID-T2DM represents the strongest marker associated with higher adverse in-hospital outcomes. It is predominantly operational in patients undergoing PN. In consequence, patients undergoing ID-T2DM PN may represent a particularly relevant target population for optimization to reduce the observed detrimental effects. Tailored prehabilitation, minimized fasting periods and enhanced perioperative monitoring have been suggested as feasible and effective approaches.^25–27^ Given the lower numbers of PN relative to RN and that patients with ID-T2DM represent a relatively small subgroup in patients undergoing nephrectomy, the target population is reduced from a total of 21,394 patients with T2DM to 1109 patients with ID-T2DM undergoing PN. Only a limited number of high-risk individuals need to be identified and managed to potentially prevent a meaningful number of complications, which renders such optimization efforts economical feasible. Similar considerations, that are based on less robust observations, may be contemplated in patients with ID-T2DM undergoing RN. Finally, NID-T2DM appears unlikely to warrant population-level health interventions in patients undergoing nephrectomy, given its weak association with adverse in-hospital outcomes.

Several limitations require acknowledgement. First, our study shares the limitations of all similar studies that were based on the NIS database and relied on a retrospective data analysis. The reliance on administrative coding may introduce potential biases, including misclassification of diagnoses and procedures. Second, the lack of data on tumor size, stage, and grade, as well as operative details such as ischemia time, anatomical complexity, and surgeon or institutional volume, limits our ability to adjust for oncologic or technical factors that may influence perioperative risk and introduces the potential for residual confounding. Third, detailed T2DM metrics, including HbA1c levels, are not available. A continuously coded HbA1c variable would have enabled a more granular assessment. However, the NIS provides T2DM classification in categorical form only, limiting such analysis. Fourth, insulin-dependence should be interpreted as a surrogate marker of disease severity rather than a direct causal factor. In addition to reflecting greater disease severity or metabolic instability, insulin use may also capture disease duration, renal dysfunction, socioeconomic factors, or evolving treatment practices. As such, the observed associations reflect risk stratification within the constraints of an observational study design and cannot establish causality. Moreover, the pronounced temporal increase in insulin-dependent T2DM observed in this study may in part reflect changes in coding practices and documentation over time, including the transition from ICD-9 to ICD-10, rather than true epidemiologic shifts alone. Finally, the present analysis involved multiple statistical comparisons, which may increase the risk of type I error. However, the main conclusions are not based on isolated statistically significant findings, but on consistent patterns characterized by robust signals across several adverse in-hospital outcomes, with differences primarily reflecting the magnitude of the associations.

## Supplementary Information

Below is the link to the electronic supplementary material.

Supplementary file1 (DOCX 22 KB)
