# Supplementary material for: Type 2 Diabetes Mellitus Versus Adverse In-Hospital Outcomes After Partial or Radical Nephrectomy
Source: Ann Surg Oncol. 2026 Feb 27;33(6):5874–83. doi: 10.1245/s10434-026-19225-9 (PMC13179263; doi:10.1245/s10434-026-19225-9)
Supplement: Supplementary file 1 — Supplementary file1 (DOCX 22 KB) [file 10434_2026_19225_MOESM1_ESM.docx]

**Table S1:** Descriptive characteristics of patients undergoing partial or radical nephrectomy, stratified according to type 2 diabetes mellitus and insulin dependence, after propensity score matching.

| **Characteristic** | **Partial Nephrectomy** | | | | | | **Radical Nephrectomy** | | | | | |
| --- | --- | --- | --- | --- | --- | --- | --- | --- | --- | --- | --- | --- |
|  | **ID-T2DM patients**  **n = 1,109 (100%)** | **non-diabetic patients**^1^  **n = 5,545 (22.7% after 1:5 PSM)** | **p-value**^2^ | **NID-T2DM patients**  **n = 6,400 (100%)** | **non-diabetic patients**^1^  **n = 6,400 (26.2% after 1:1 PSM)** | **p-value**^2^ | **ID-T2DM patients**  **n = 1,961 (100%)** | **non-diabetic patients**^1^  **n = 3,922 (9.1% after 1:2 PSM)** | **p-value**^2^ | **NID-T2DM patients**  **n = 11,924 (100%)** | **non-diabetic patients**^1^  **n = 11,924 (27.6% after 1:1 PSM)** | **p-value**^2^ |
| **Age**, median (IQR), in years | 62 (56, 69) | 63 (55, 70) | 0.2 | 63 (57, 70) | 64 (57, 70) | 0.2 | 65 (57, 71) | 65 (57, 72) | 0.6 | 66 (58, 73) | 66 (58, 73) | 0.6 |
| **Male sex**, n (%) | 654 (59.0%) | 3,304 (59.6%) | 0.7 | 3,929 (61.4%) | 4,000 (62.5%) | 0.2 | 1,169 (59.6%) | 2,349 (59.9%) | 0.8 | 7,431 (62.3%) | 7,390 (62.0%) | 0.6 |
| **Ethnicity**, n (%) |  |  | 0.9 |  |  | 0.7 |  |  | 0.9 |  |  | 0.9 |
| Caucasian | 740 (66.7%) | 3,704 (66.8%) |  | 4,442 (69.4%) | 4,456 (69.6%) |  | 1,267 (64.6%) | 2,544 (64.9%) |  | 8,479 (71.1%) | 8,491 (71.2%) |  |
| African American | 181 (16.3%) | 930 (16.8%) |  | 835 (13.0%) | 856 (13.4%) |  | 341 (17.4%) | 685 (17.5%) |  | 1,396 (11.7%) | 1,410 (11.8%) |  |
| Hispanic | 124 (11.2%) | 616 (11.1%) |  | 678 (10.6%) | 673 (10.5%) |  | 245 (12.5%) | 477 (12.2%) |  | 1,340 (11.2%) | 1,324 (11.1%) |  |
| Other | 64 (5.8%) | 295 (5.3%) |  | 445 (7.0%) | 415 (6.5%) |  | 108 (5.5%) | 216 (5.5%) |  | 709 (5.9%) | 699 (5.9%) |  |
| **Charlson Comorbidity Index**^3^, n (%) |  |  | 0.2 |  |  | 0.6 |  |  | 0.9 |  |  | 0.4 |
| 0-1 | 695 (62.7%) | 3,597 (64.9%) |  | 4,754 (74.3%) | 4,779 (74.7%) |  | 1,024 (52.2%) | 2,046 (52.2%) |  | 8,068 (67.7%) | 8,123 (68.1%) |  |
| ≥2 | 414 (37.3%) | 1,948 (35.1%) |  | 1,646 (25.7%) | 1,621 (25.3%) |  | 937 (47.8%) | 1,876 (47.8%) |  | 3,856 (32.3%) | 3,801 (31.9%) |  |
| **Obesity**^4^, n (%) | 421 (38.0%) | 1,956 (35.3%) | 0.1 | 1,883 (29.4%) | 1,835 (28.7%) | 0.4 | 698 (35.6%) | 1,394 (35.5%) | 0.9 | 3,045 (25.5%) | 3,054 (25.6%) | 0.9 |
| **Year of surgery**, median (IQR) | 2016  (2012, 2018) | 2016  (2012, 2018) | 0.1 | 2014  (2010, 2017) | 2014  (2010, 2017) | 0.9 | 2015  (2011, 2017) | 2015  (2011, 2017) | 0.2 | 2012  (2008, 2016) | 2012  (2008, 2016) | 0.9 |
| **Minimally invasive procedure**, n (%) | 608 (54.8%) | 3,136 (56.6%) | 0.3 | 3,118 (48.7%) | 3,215 (50.2%) | 0.1 | 754 (38.4%) | 1,437 (36.6%) | 0.2 | 3,651 (30.6%) | 3,747 (31.4%) | 0.2 |
| **Hospital size**^5^, n (%) |  |  | 0.6 |  |  | 0.6 |  |  | 0.7 |  |  | 0.7 |
| Large | 729 (65.7%) | 3,596 (64.9%) |  | 4,293 (67.1%) | 4,238 (66.2%) |  | 1,235 (63.0%) | 2,508 (63.9%) |  | 7,698 (64.6%) | 7,739 (64.9%) |  |
| Medium | 241 (21.7%) | 1,283 (23.1%) |  | 1,384 (21.6%) | 1,426 (22.3%) |  | 492 (25.1%) | 950 (24.2%) |  | 2,834 (23.8%) | 2,833 (23.8%) |  |
| Small | 139 (12.5%) | 666 (12.0%) |  | 723 (11.3%) | 736 (11.5%) |  | 234 (11.9%) | 464 (11.8%) |  | 1,392 (11.7%) | 1,352 (11.3%) |  |
| ^1^PSM relied on patient age, sex, ethnicity, Charlson Comorbidity Index, obesity status, minimally invasive procedure and year of surgery. ^2^Wilcoxon rank-sum test, Pearson's chi-squared test, Reference: matched non-diabetic patients. ^3^The point contribution of CCI due to T2DM was subtracted. ^4^Body mass index > 30 kg/m^2^. ^5^Defined by bed count; specific to region, urban or rural location and teaching-hospital status.  Abbreviations: ID = Insulin-dependent, IQR = interquartile range, NID = Non-insulin-dependent, PSM = propensity score matching, T2DM = type 2 diabetes mellitus. | | | | | | | | | | | | |
